# Supplementary material for: Novel Virulent Bacteriophage ΦSG005, Which Infects Streptococcus gordonii, Forms a Distinct Clade among Streptococcus Viruses
Source: Viruses. 2021 Sep 29;13(10):1964. doi: 10.3390/v13101964 (PMC8537203; doi:10.3390/v13101964)
Supplement: Supplementary file 1 [file viruses-13-01964-s001.zip › viruses-1352664-supplementary.pdf]

**Supplementary Materials**

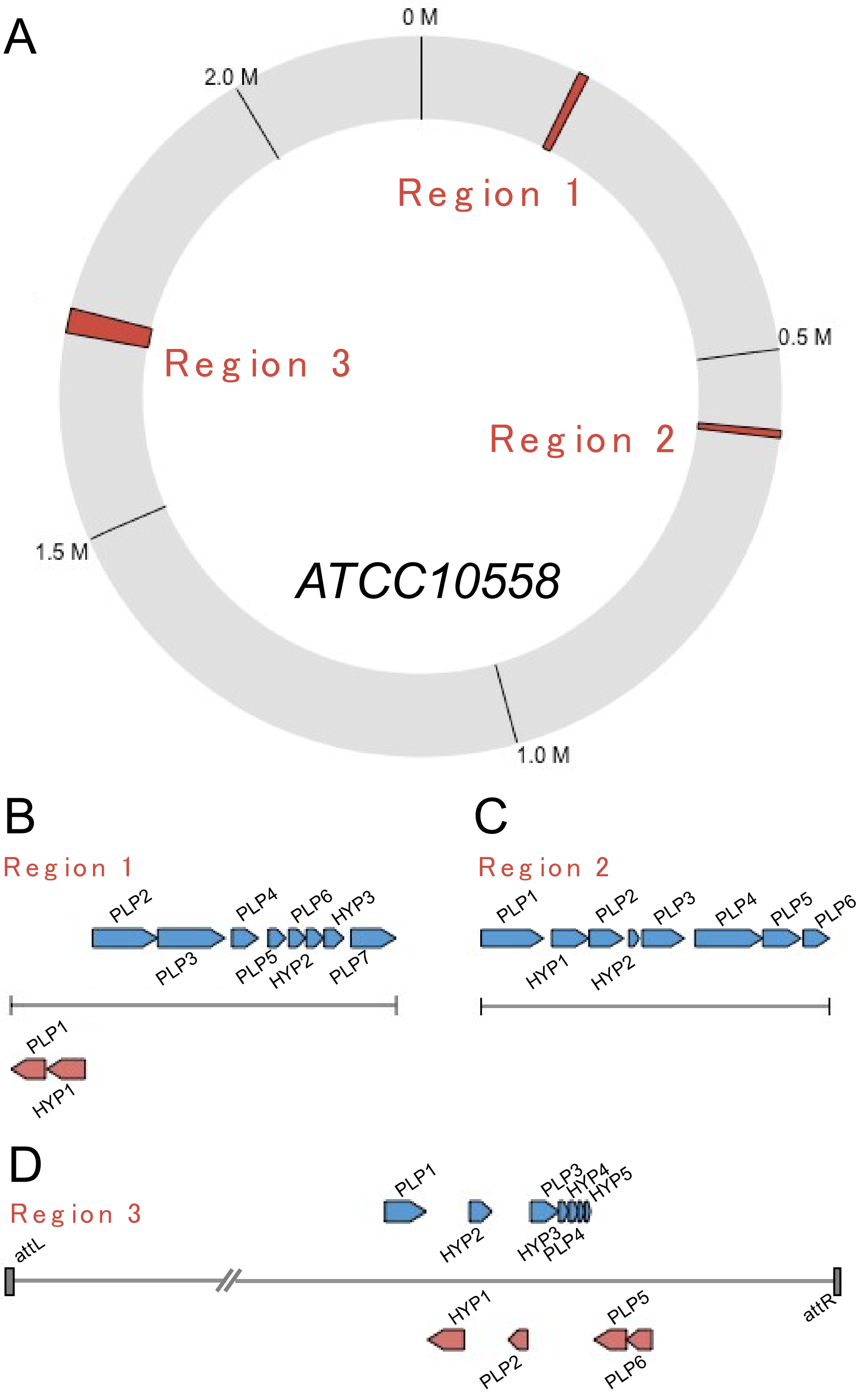


**Figure S1.** Identification of prophage-associated regions in S. gordonii ATCC10558.

(A) Three prophage-associated regions were detected in the chromosome of *S. gordonii* ATCC10558 by PHASTER analysis. (B-D) Genome structure of each region. Inferred CDSs are indicated as blue or red arrows (complementary).

**Table S1.** Products and predicted functions of proteins encoded by each region described in Fig. S1B-D.

| Rea-gion | Completeness | Location (nucleotides) | G+C (%) | Gene name | CDS Location (nucleotides) | Str-and | BLAST Hit | |
|---|---|---|---|---|---|---|---|---|
| 1 | incomplete | 157064-167169 | 43.2 | PLP1 | 157064-157966 | - | hypothetical protein | NC_031245 |
| | | | | PLP2 | 159207-160913 | + | ABC transporter | NC_001884 |
| | | | | PLP3 | 160915-162684 | + | ABC transporter | NC_001884 |
| | | | | PLP4 | 162863-163552 | + | putative amidotransferase | NC_027988 |
| | | | | PLP5 | 163816-164265 | + | bifunctional NMN adenylyltransferase/nudix hydrolase | NC_023568 |
| | | | | PLP6 | 164350-164793 | + | hypothetical protein | NC_031017 |
| | | | | PLP7 | 165976-167169 | + | hypothetical protein | NC_047958 |
| | | | | HYP1 | 157996-159012 | - | | |
| | | | | HYP2 | 164803-165261 | + | | |
| | | | | HYP3 | 165280-165795 | + | | |
| 2 | incomplete | 579068-586553 | 41.2 | PLP1 | 579068-580411 | + | deoxynucleoside kinase | NC_041884 |
| | | | | PLP2 | 581390-582133 | + | ABC transporter | NC_016564 |
| | | | | PLP3 | 582527-583441 | + | gp344 | NC_023719 |
| | | | | PLP4 | 583664-585124 | + | gp277 | NC_023719 |
| | | | | PLP5 | 585121-585945 | + | gp283 | NC_023719 |
| | | | | PLP6 | 585993-586553 | + | putative acetyltransferase | NC_016564 |
| | | | | HYP1 | 580581-581390 | + | | |
| | | | | HYP2 | 582239-582463 | + | | |
| 3 | incomplete | 1703318-1727504 | 44.7 | attL | 1703318-1703332 | + | | |
| | | | | attR | 1727504-1727518 | + | | |
| | | | | PLP1 | 1716456-1717460 | + | hypothetical protein | NC_042057 |
| | | | | PLP2 | 1719466-1719939 | - | hypothetical protein | NC_047924 |
| | | | | PLP3 | 1720012-1720707 | + | hypothetical protein | NC_042128 |
| | | | | PLP4 | 1721164-1721325 | + | hypothetical protein | NC_048737 |
| | | | | PLP5 | 1721550-1722350 | - | gp245 | NC_023719 |
| | | | | PLP6 | 1722337-1722960 | - | ABC transporter | NC_016564 |
| | | | | HYP1 | 1717494-1718384 | - | | |
| | | | | HYP2 | 1718517-1719071 | + | | |
| | | | | HYP3 | 1720700-1720936 | + | | |
| | | | | HYP4 | 1720949-1721176 | + | | |
| | | | | HYP5 | 1721340-1721468 | + | | |
